# Supplementary material for: Apicobasal RNA asymmetries regulate cell fate in the early mouse embryo
Source: Nat Commun. 2023 May 30;14:2909. doi: 10.1038/s41467-023-38436-2 (PMC10229589; doi:10.1038/s41467-023-38436-2)
Supplement: Supplementary file 1 — Supplementary Information [file 41467_2023_38436_MOESM1_ESM.pdf]

Apicobasal RNA asymmetries regulate cell fate in the early mouse embryo

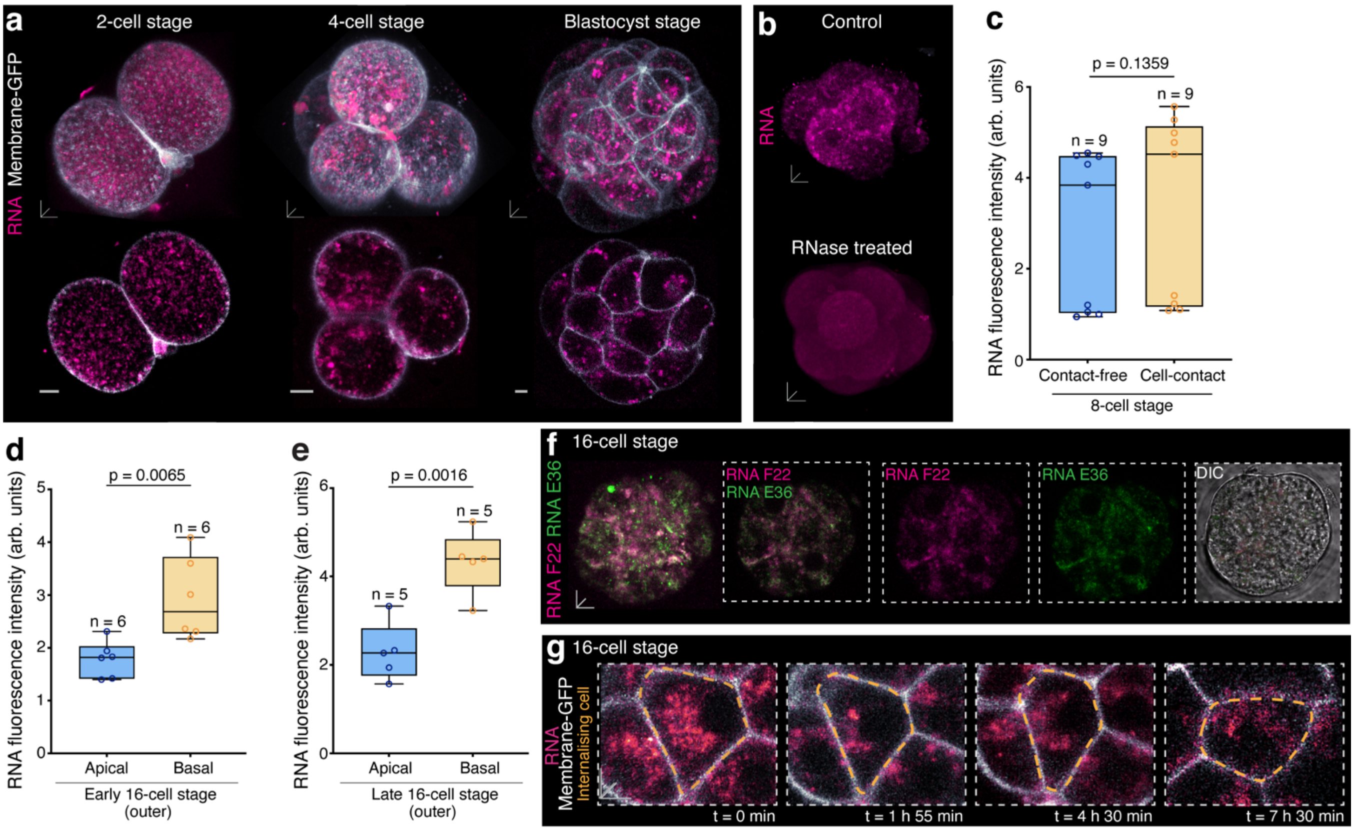

Supplementary Fig. 1 RNA localisation in preimplantation mouse embryos.
a RNA localisation in 2-cell, 4-cell and blastocyst stage embryos. 3D (top) and 2D (bottom) view. **b** Validation of RNA dye specificity by RNase treatment. **c** Quantification of RNA fluorescence intensity in cell contact-free and cell-cell contact regions at 8-cell stage. RNA fluorescence intensity in apical and basal regions of **d**, early and **e**, late 16-cell stage outer blastomeres. **f** Co-labelling of 16-cell stage preimplantation mouse embryo with E36 and F22 RNA dye. **g** RNA localisation in inner blastomere (orange dashed line) throughout 16-cell stage. Box plots display minimum, lower quartile, median, upper quartile and maximum. Individual data points are overlaid. Mann-Whitney two-tailed t-tests were used to identify statistical differences. Scale bars 10 µm; insets 5 µm. Source data are provided as a Source data file.

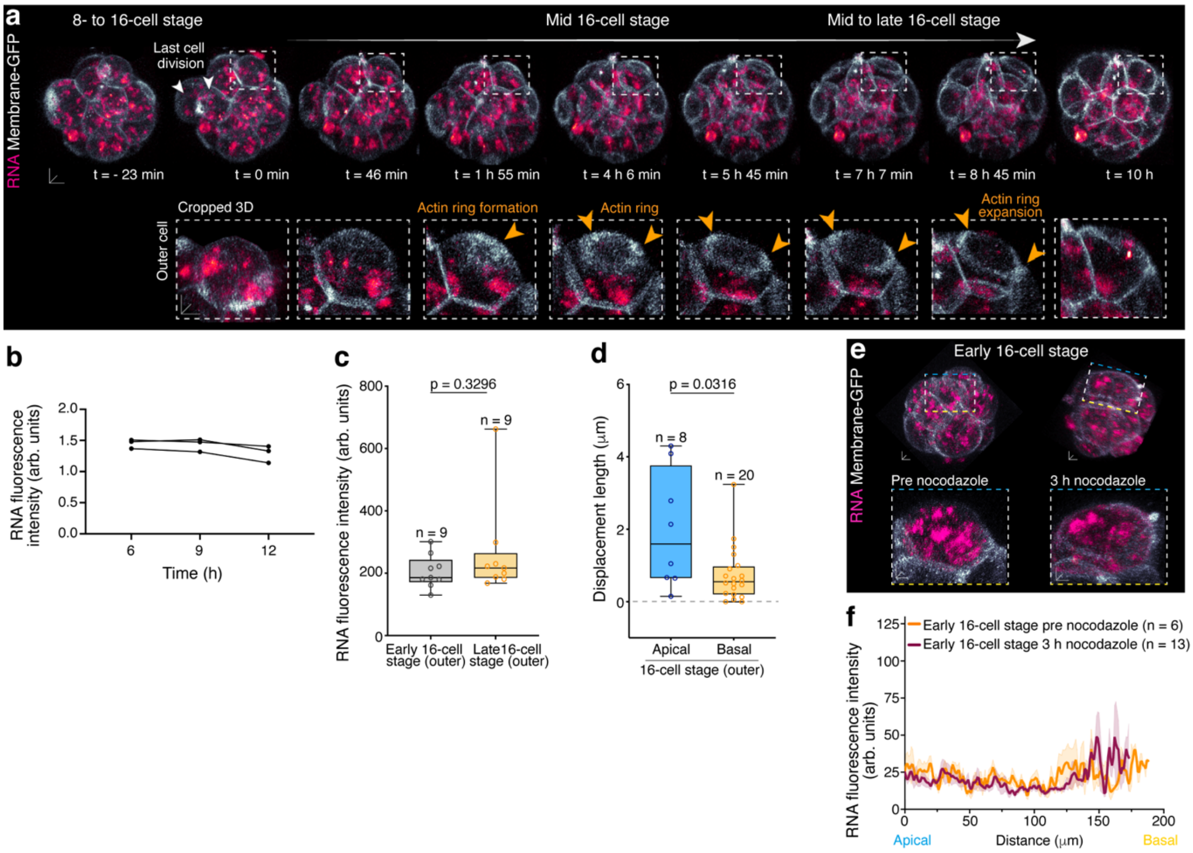

Supplementary Fig. 2 Polarised microtubule cytoskeleton directs RNA transport.
a Time-lapse imaging of 3D embryo (top) throughout the 16-cell stage interphase reveals RNA localisation towards basal regions accompanying actin ring expansion (orange arrowheads in cropped single blastomere (bottom insets)). **b** RNA fluorescence intensity at the basal membrane of outer blastomeres from mid- to late 16-cell stage; data shows individual values. **c** Total RNA intensity of outer blastomeres in early and late 16-cell stage embryos. **d** RNA displacement length in apical and basal regions of late 16-cell stage embryos. **e** Early 16-cell stage embryos untreated and following 3 h nocodazole treatment. Cropped single blastomeres shown (insets). **f** Quantification of RNA fluorescence intensity from apical to basal regions in **e**; Data are presented as mean ± SEM; thick lines indicate mean, transparent shadows depict SEM. Box plots display minimum, lower quartile, median, upper quartile and maximum. Individual data points are overlaid. Mann-Whitney two-tailed t-tests were used to identify statistical differences. Scale bars 10 µm; insets 5 µm. Source data are provided as a Source data file.

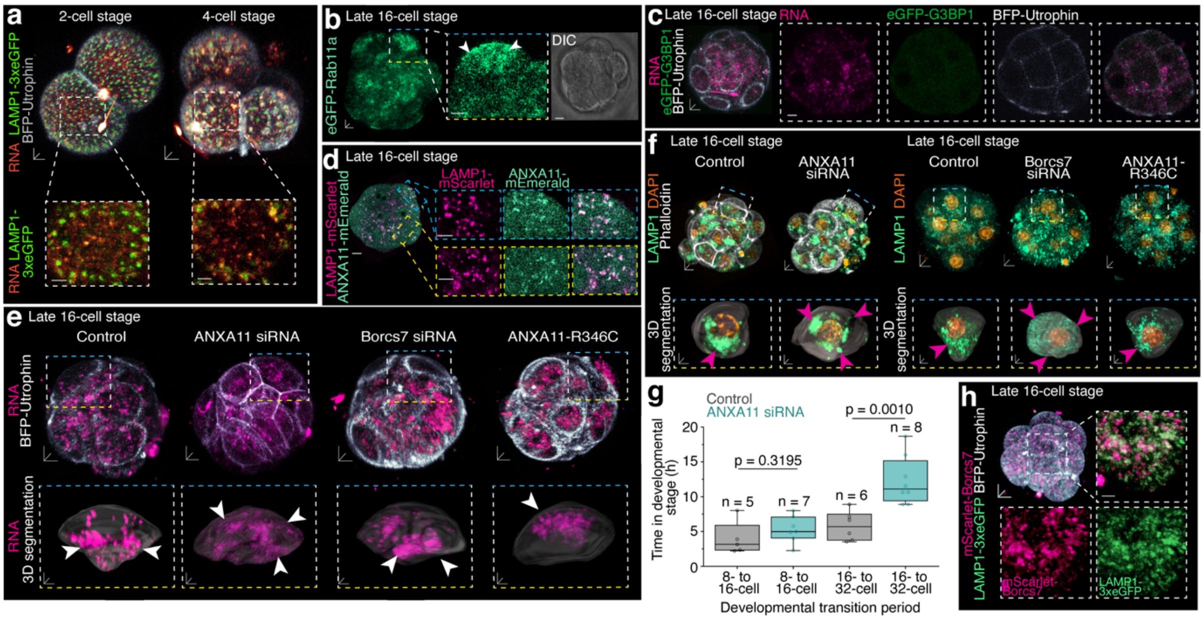

Supplementary Fig. 3 RNA trafficking requires the tripartite complex of microtubules, lysosomes and Annexin A11.

a Spatial distribution of RNA and lysosomes (LAMP1-3xeGFP) at 2-cell and 4-cell stage in 3D embryos and 2D planes through cytoplasmic region (insets). **b** Rab11a (eGFP-Rab11a) localisation (white arrowheads) at late 16-cell stage. **c** Live imaging of G3BP1 (eGFP-G3BP1), RNA and BFP-Utrophin shows absence of RNA stress granule maker eGFP-G3BP1 in late 16-cell stage embryos. **d** Live imaging of late 16-cell stage embryo labelled with Annexin A11 (ANXA11-mEmerald) and lysosomes (LAMP1-mScarlet). Insets show apical (blue dashed boxes) and basal (yellow dashed boxes) view. **e** RNA localisation (white arrowheads) in control, Annexin A11 (ANXA11) siRNA-, Borcs7 siRNA- and ANXA11-R346C-mutant embryos, segmented and masked single blastomere shown (insets). **f** Asymmetric localisation of LAMP1-positive vesicular-like structures (pink arrowheads) is disrupted in Annexin A11 siRNA- and Borcs7 siRNA-treated embryos, but not in ANXA11-R346C mutant embryos. Segmented and masked single blastomere shown (insets). **g** Developmental rate of Annexin A11 knockdown embryos compared to control embryos. Box plot displays minimum, lower quartile, median, upper quartile and maximum. Individual data points are overlaid. n = number of embryos. Welch’s two-tailed unpaired t-tests were used to identify statistical differences. **h** Lysosomal subunit Borcs7 (mScarlet-Borcs7) is located with lysosomes (LAMP1-3xeGFP) in late 16-cell stage embryos. Scale bars 10 µm; insets 5 µm. Source data are provided as a Source data file.

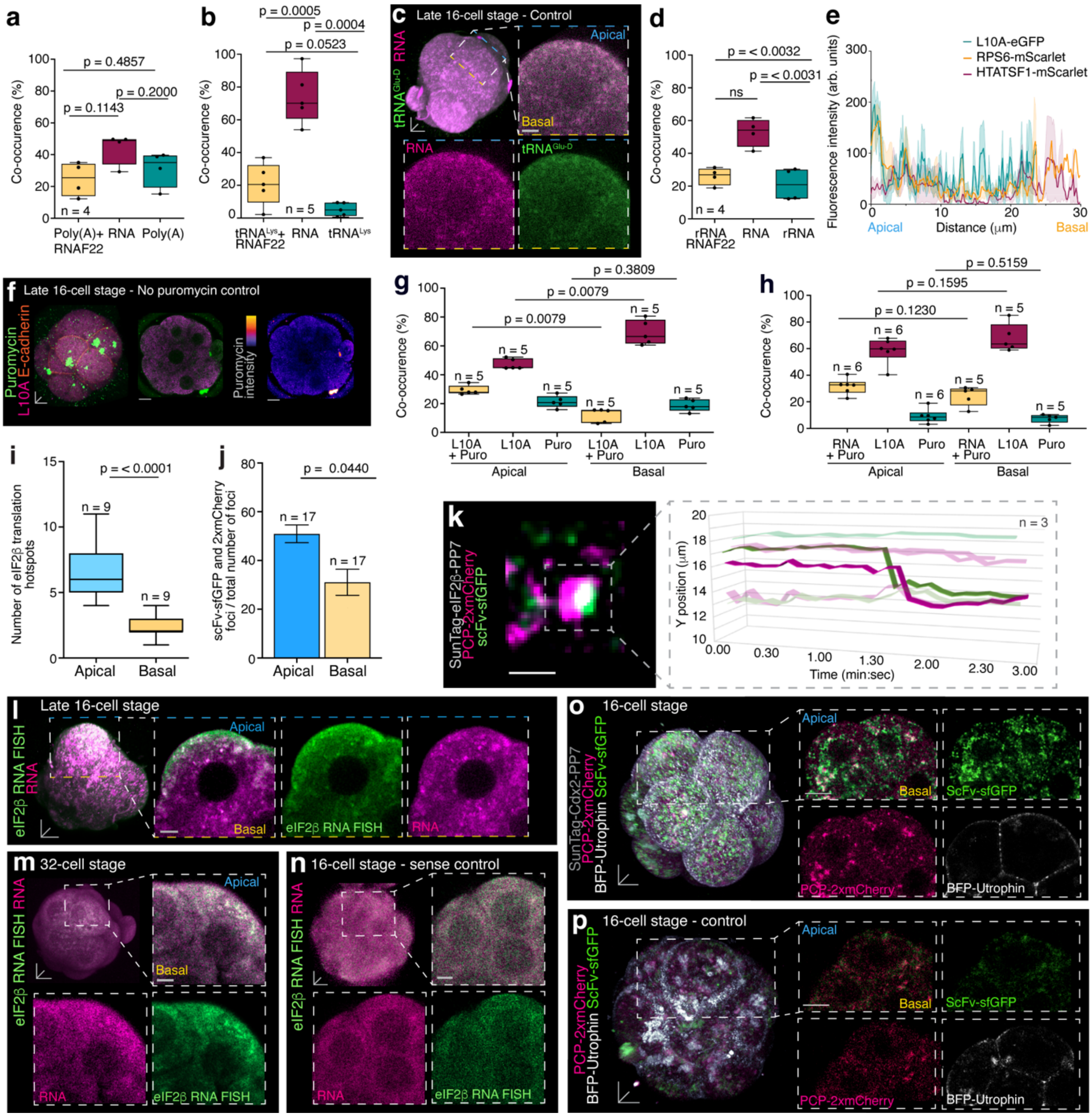

Supplementary Fig. 4 Higher translational activity of apical RNA transcripts.

a Quantification of mRNA (Poly(A) RNA FISH) and RNA in late 16-cell stage embryos shows percentage of pixel co-occurrence, only RNA or only Poly(A)FISH signal. **b** Quantification of tRNA FISH and RNA pixel co-occurrence. **c** tRNA FISH negative control against *Dictyostelium discoidium* tRNA$^{Glu\text{-}D}$. **d** Quantification of ribosomal RNA (rRNA-naphthalimide) and RNA pixel co-occurrence. **e** Fluorescence intensity quantification of large 60S ribosomal subunit (L10A-eGFP), small ribosomal 40S subunit RPS6 (RPS6-mScarlet) and RNA-binding protein HIV-1 Tat Specific Factor 1 (HTATSF1-mScarlet) in apical and basal regions of late 16-cell stage embryos; data are presented as mean ± SEM; thick lines indicate mean, transparent shadows depict SEM. **f** Ribopuromycylation method (RPM); control experiment without puromycin shows absence of puromycylated ribosome-bound nascent chains. **g** Pixel co-occurrence quantification of **f**. **h** Quantification of pixel co-occurrence in late 16-cell stage embryos following RPM, labelled with RNA and Puromycin. **i** Quantification of number of eIF2β translation hotpots in apical and basal regions of late 16-cell stage embryo

using SunTag eIF2β. **j** Ratio of scFv-sfGFP and 2xmCherry-labelled foci in apical and basal regions of late 16-cell stage embryo; data presented as mean ± SEM. **k** SunTag system visualising eIF2β translation dynamics, faint lines show replicates, bold lines indicate mean. **l** RNA FISH for eIF2β at late 16-cell stage, **m** at 32-cell stage and **n** sense control. **o**, **p** Cdx2 and control SunTag reporter in live 16-cell stage embryos. Box plots in a, b, d and g-i display minimum, lower quartile, median, upper quartile and maximum. Individual data points are overlaid Mann-Whitney or Welch's two-tailed unpaired t-tests were used to identify statistical differences. Scale bars 10 μm; insets 5 μm; except inset in k 1 μm. Source data are provided as a Source data file.

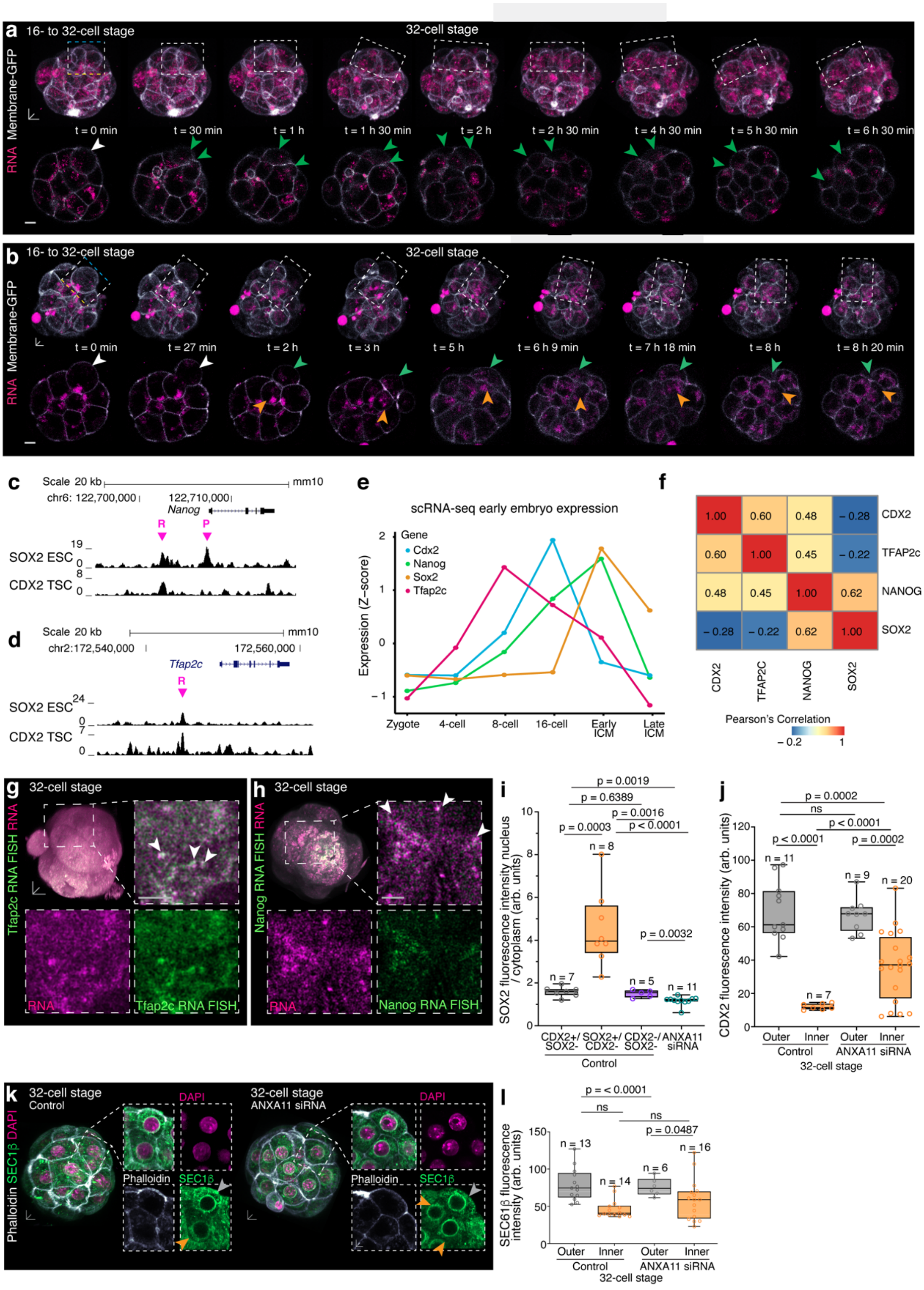
a
16- to 32-cell stage
32-cell stage
RNA Membrane-GFP
t = 0 min
t = 30 min
t = 1 h
t = 1 h 30 min
t = 2 h
t = 2 h 30 min
t = 4 h 30 min
t = 5 h 30 min
t = 6 h 30 min
b
16- to 32-cell stage
32-cell stage
RNA Membrane-GFP
t = 0 min
t = 27 min
t = 2 h
t = 3 h
t = 5 h
t = 6 h 9 min
t = 7 h 18 min
t = 8 h
t = 8 h 20 min
c
Scale 20 kb
mm10
chr6: 122,700,000
122,710,000
Nanog
R
P
SOX2 ESC
CDX2 TSC
19
0
8
0
d
Scale 20 kb
mm10
chr2:172,540,000
172,560,000
Tfap2c
R
SOX2 ESC
CDX2 TSC
24
0
7
0
e
scRNA-seq early embryo expression
Gene
Cdx2
Nanog
Sox2
Tfap2c
Expression (Z-score)
2
1
0
− 1
Zygote
4-cell
8-cell
16-cell
Early ICM
Late ICM
f
1.00
0.60
0.48
− 0.28
CDX2
0.60
1.00
0.45
− 0.22
TFAP2c
0.48
0.45
1.00
0.62
NANOG
− 0.28
− 0.22
0.62
1.00
SOX2
CDX2
TFAP2C
NANOG
SOX2
Pearson's Correlation
− 0.2
1
g
32-cell stage
Tfap2c RNA FISH RNA
RNA
Tfap2c RNA FISH
h
32-cell stage
Nanog RNA FISH RNA
RNA
Nanog RNA FISH
i
p = 0.0019
p = 0.6389
p = 0.0016
p = 0.0003
p < 0.0001
p = 0.0032
n = 8
n = 7
n = 5
n = 11
SOX2 fluorescence intensity nucleus / cytoplasm (arb. units)
10
8
6
4
2
0
CDX2+/ SOX2-
SOX2+/ CDX2-
CDX2-/ SOX2-
ANXA11 siRNA
Control
j
p = 0.0002
ns
p < 0.0001
p < 0.0001
p = 0.0002
n = 11
n = 9
n = 20
n = 7
CDX2 fluorescence intensity (arb. units)
120
100
80
60
40
20
0
Outer
Inner
Outer
Inner
Control
ANXA11 siRNA
32-cell stage
k
32-cell stage
Control
32-cell stage
ANXA11 siRNA
Phalloidin SEC1β DAPI
DAPI
Phalloidin
SEC1β
l
p = < 0.0001
ns
ns
p = 0.0487
n = 13
n = 14
n = 6
n = 16
SEC61β fluorescence intensity (arb. units)
150
100
50
0
Outer
Inner
Outer
Inner
Control
ANXA11 siRNA
32-cell stage

Supplementary Fig. 5 RNA and translation component asymmetries contribute to cell fate.

a Tracking of cell position following symmetric cell division (white arrowhead) from 16- to 32-cell stage. Bottom panels show 2D planes with tracked outer daughter blastomeres (green arrowheads). **b** Tracking of cell position following asymmetric cell division (white arrowhead) from 16- to 32-cell stage. Bottom panels show 2D planes with tracked inner (orange arrowhead) and outer blastomere (green arrowhead). **c** Binding profiles of SOX2 and CDX2 at the potential target genes NANOG and **d** TFAP2C, with pink arrows indicating binding at a regulatory element (R) or promoter (P) region. **e** Expression profiles of Sox2, Cdx2, and two of their potential targets, Nanog and Tfap2c, throughout early embryonic development. **f** Pairwise Pearson's correlation between all four genes throughout early embryogenesis. **g** RNA FISH for Tfap2c in 32-cell stage embryo and **h** RNA FISH for Nanog in 32-cell stage embryo; white arrowheads indicate co-localisation of RNA foci with RNA FISH signal. **i** Nuclear to cytoplasmic SOX2 fluorescence intensity ratio reveals absence of SOX2-postive nuclei following Annexin A11 knockdown (ANXA11 siRNA). **j** Quantification of CDX2 fluorescence intensity in outer and inner blastomeres of control and Annexin A11 siRNA-treated embryos. **k** SEC61β fluorescence intensity in inner (orange arrowheads) and outer blastomeres (grey arrowheads) of Annexin A11 siRNA-treated 32-cell stage embryos compared to controls. **l** Quantification of **e**. Box plots in i, j and l display minimum, lower quartile, median, upper quartile and maximum. Individual data points are overlaid. Mann-Whitney or Welch's two-tailed unpaired t-tests were used to identify statistical differences. Scale bars 10 µm; 2D panels 10 µm; insets 5 µm, except insets in g, h and k 5 µm. Source data are provided as a Source data file.

Table S1. Primer and siRNA sequences

Primer name	Sequence (5' to 3')
3xeGFP_Frag_Fwd	caaggatccggtcgccaccatg
3xeGFP_Frag_Rev	acGTAATACGatggaatcctggcttagaatt
ANXA11_Frag_Fwd	CTCTAGAACTatgagctaccctggctatccc
ANXA11_Frag_Rev	CGACTCACTATttacttgtacagctcgtccatgcc
ANXA11_Vector_Fwd	tgtacaagtaaATAGTGAGTCGTATTACGTAGATCCAGACA
ANXA11_Vector_Rev	gggtagctcatAGTTCTAGAGGCTCGAGAGGC
ANXA11-R346C _Frag_Fwd	TATAGAATACAaccatgagctaccctggct
ANXA11-R346C - Vector_REV	gtagctcatggtTGTATTCTATAGTGTCACCTAAATCAAGCTT GCTCC
ANXA11-R346C Frag_Rev	ATCTTATCATGTttacttgtacagctcgtccatgcc
ANXA11-R346C_Vector.FWD	ctgtacaagtaaACATGATAAGATACATTGATGAGTTTGGAC AAAC
Cdx2-SunTag Vector_Fwd	tgaaagcttcacgattcgaagatcgcatcgc
Cdx2-SunTag Vector_Rev	CTCACGTAtgaccggtgcggc
Cdx2-SunTag_Frag_Fwd	accggtcaTACGTGAGCTACCTTCTGGACAAG
Cdx2-SunTag_Frag_Rev	cgaatcgtgaagctttcaCTGGGTGACAGTG
eIF2β-SunTag _Fwd	ggctcgggtcagcggccgcaaagcttcgaattcatgtc
eIF2β-SunTag _Rev	tacctgtacaaccgttaggatccgttagctttggc
G3BP1_Frag_Fwd	tcagatccgtgatggagaagcctagtcccc
G3BP1_Frag_Rev	TTCTAGAGGtcactgccgtggcgca
G3BP1_Vector_Fwd	ccacggcagtgaCCTCTAGAACTATAGTGAGTCGTATT
G3BP1_Vector_Rev	gactaggcttctccatcacggatctgagtcc
HTATSF1_Frag_Fwd	CTACTTGTatgagcggcaccaacctg
HTATSF1_Frag_Rev	cgatcgctcttgtcatcgtcatccttgtagtcgaa
HTATSF1_Vector_Fwd	atgacaagagcgatcgccggaCA
HTATSF1_Vector_Rev	gccgctcatACAAGTAGCTTGTATTCTATAGTGTCACCTAA ATCA
Kif18b-SunTag Frag_Rev	catggtggcAGTTCTAGAGGCTCGAGAGGC
Kif18b-SunTag Vector_Rev	TAAATCAAGCTaacgcgtatatctggccc
Kif18b-SunTag_Frag_Fwd	tatacgcgttAGCTTGATTTAGGTGACACTATAGAATACAA GC
Kif18b-SunTag_Vector_Fwd	CTAGAACTgccaccatgggcg
L10A_Frag_Fwd	GCAGGATCatgagcagcaaagtttcacgc
L10A_Frag_Rev	ATGtccggcggctcactcctcctcctc
L10A_Vector_Fwd	agtgagccgccggaCATCGATTatgg
L10A_Vector_Rev	ctgctcatGATCCTGCAAAAAGAACAAGTAGCTTGT
LAMP1_Vector_Fwd	CTCTAGAACTatgcccaaaaagaagaggaaagtgaac
LAMP1_Vector_Rev	CTCACTATtcgggcgagacgctatt
mEmerald-Sec61β_Frag_Fwd	TTCTAGAGGctacgaacgagtgtacttgccc
mEmerald-Sec61β_Frag_Rev	AATACAAGCatggtgagcaagggcgag
mEmerald-Sec61β_Vector_Fwd	cgttcgtagCCTCTAGAACTATAGTGAGTCGTATTACG
mEmerald-Sec61β_Vector_Rev	gctcaccatGCTTGTATTCTATAGTGTCACCTAAATCAAGC T
mScarlet-Borcs7_Frag_Fwd	TTGTTCTTgtcgccaccatggtgagc
mScarlet-Borcs7_Frag_Rev	TATCATGTCTGctacttcagtaagtgactcaactgg

mScarlet-Borcs7_Vector_Fwd	tactgaagtagCAGACATGATAAGATACATTGATGAGTTTGGACA
mScarlet-Borcs7_Vector_Rev	gtggcgacAAGAACAAGTAGCTTGTATTCTATAGTGTCACCTAAATCA
PABPC1_Frag_Fwd	ACAAGCTACTatggcttctaactttactcagttcgttct
PABPC1_Frag_Rev	tagaagccatAGTAGCTTGTATTCTATAGTGTCACCTAAATCAAG
PABPC1_Vector_Fwd	tccaactgttTCGATTatggtgagcaagggc
PABPC1_Vector_Rev	ccatAATCGAaacagttggaacaccggtg
PCP-2xmCherry_Frag_Fwd	ttttggacatggtAGTTCTAGAGGCTCGAGAgg
PCP-2xmCherry_Frag_Rev	GACTCACTATaccagaacctccacccatatgc
PCP-2xmCherry_Vector_Fwd	tggtATAGTGAGTCGTATTACGTAGATCCAGACATGATAAGa
PCP-2xmCherry_Vector_Rev	ttttggacatggtAGTTCTAGAGGCTCGAGAgg
peGFP_SP6_Fwd	ATTTAGGTGACACTATAGAAGAACCGTCAGATCCGCTAG
peGFP_SV40_Rev	CTCAACCCTATCTCGGTCTATTCT
RPS6-mScarlet_Frag_Fwd	ctgtacaagatagggagacccaagcttCTTTTCC
RPS6-mScarlet_Frag_Rev	cctttaattaaagtggatccTTTCTGACTGGATTCAG
RPS6-mScarlet_Vector_Fwd	AAggatccactttaattaaaggccggccagcg
RPS6-mScarlet_Vector_Rev	gtctccctatcttgtacagctcgtccatgcc
eIF2β SP6 RiboProbe_Fwd	tatatATTTAGGTGACACTATAGGTTAGCTTTGGCACGGAGCT
eIF2β RiboProbe_Rev	ATGTCCGGGGACGAGATGATTTT
Nanog SP6 RiboProbe_Fwd	tatatATTTAGGTGACACTATAGtcatatttcacctggtggagtcacag
Nanog RiboProbe_Rev	atgagtgtgggtcttcctggtc
Tfap2c Sp6 RiboProbe_Fwd	tatatATTTAGGTGACACTATAGttacttcctgtgcttttccattttctcca
Tfap2c RiboProbe_Rev	ttcgccatgttgtggaaaataacagataatg
siRNA targeting Annexin A11 ANXA11-1 (Qiagen)	AAGGATTTGATCAAAGACCTA
siRNA targeting Borcs7 (Qiagen)	CAGGACCAGTTGAGTCACTTA
mature rat $tRNA^{LysCUU}$	CCA ACG TGG GGC TCG AAC CCA CGA CCC TGA GAT TAA GAG TCT CAT GCT CTA CCG ACT /3FluorT/
D. discoidium $tRNA^{Glu}$	CCA GTG TTA GAG ACT AGA GTG TAC CGA CTA CAC CAA TGA /3FluorT/
Oligo(dT)-18-5FluorT	TTT TTT TTT TTT TTT TTT / 5FluorT /

Table S2. Plasmids

Reagent / resource	Source
FUW-TetO-Tfap2c	Addgene #128826
LAMP1-mScarlet-1	Addgene #98827
mScarlet-Borcs7	Addgene #118748
p.R346C-ANXA11	Addgene #164213
p323-L10A-PATagRFP	Addgene #74172[10]
pcDNA3-RPS6	Addgene #52913
pcDNA4TO_24xGCN4_v4-Kif18b-24xPP7	Addgene #74928
pCI-MS2V5-PABPC1	Addgene #65807
$pCS2^+$ ANXA11-mEmerald	This paper

pCS2$^+$ BFP-Utrophin	[9]
pCS2$^+$ eGFP-MAP2c	[3]
pCS2$^+$ eGFP-Rab11a WT	Addgene #12674
pCS2$^+$ Emerald-Sec61β	Dr. Grace Lim
pCS2$^+$ G3PB1-eGFP	This paper
pCS2$^+$ HTATSF1-B3-Scarlet	This paper
pCS2$^+$ L10A-eGFP	This paper
pCS2$^+$ LAMP1-3xeGFP	This paper
pCS2$^+$ LAMP1-mScarlet	This paper
pCS2$^+$ Membrane-Cerulean	This paper
pCS2$^+$ Membrane-GFP	This paper
pCS2$^+$ mScarlet-Borcs7	This paper
pCS2$^+$ mTFP1-Utrophin	[9]
pCS2$^+$ p.R346C-ANXA11-mEmerald	This paper
pCS2$^+$ PCP-2xmCherry	This paper
pCS2$^+$ RPS6-mScarlet	This paper
peGFP-mEmerald-Sec61β	Addgene #90992
peGFP-N1meIF2βWT	Addgene #49502
pGEM-G3BP1	Dr. Zhenghong Yuan & Prof. Derek Kennedy
pHAGE-HTATSF1-BRS3	Addgene #116822
pHR-PP7-2xmCherry-CAAX	Addgene #74925
pHR-scFV-GCN4-sfGFP-GB1-NLS-dWPRES	Addgene #60906
pLEX-EF1a ANXA11-mEmerald	Addgene #164210[9]
pMXs-Nanog	Addgene #13354
pScarlet-Borcs7	Addgene #118748
SP6-24xGCN4_v4-eIF2β-24xPP7	This paper
SP6-24xGCN4_v4-Kif18b-24xPP7	This paper
SP6-24xGCN4_v4-Cdx2-24xPP7	This paper
