# Supplementary material for: Apicobasal RNA asymmetries regulate cell fate in the early mouse embryo
Source: Nat Commun. 2023 May 30;14:2909. doi: 10.1038/s41467-023-38436-2 (PMC10229589; doi:10.1038/s41467-023-38436-2)
Supplement: Supplementary file 3 — Description of Additional Supplementary Files [file 41467_2023_38436_MOESM3_ESM.pdf]

Description of Additional Supplementary Files

File Name: Supplementary Movie 1
Description: **Asymmetric RNA localisation towards the basal membrane in outer blastomeres during 16-cell stage.** Time-lapse imaging of a live 16-cell stage mouse embryo expressing Membrane-GFP (white) and RNA (magenta) demonstrates asymmetric localisation of RNA to the basal region of outer blastomeres throughout the 16-cell interphase. Playback shows cropped 3D of a single blastomere. Time is indicated in h:m:s.

File Name: Supplementary Movie 2
Description: **Expanding polarised microtubule network directs localisation of basal RNA.** Live imaging of an early living 16-cell stage mouse embryo expressing eGFP-MAP2c (teal) and RNA (magenta) shows the expansion of the denser apical microtubule network (orange arrowheads) and simultaneous localisation of RNA foci to the basal region of outer blastomeres at late 16-cell stage. Playback shows 3D cropped cell. Time is indicated in h:m:s.

File Name: Supplementary Movie 3
Description: **Microtubule cytoskeleton guides apical-to-basal transport of RNA foci.** High temporal resolution imaging reveals RNA foci trafficking from apical (top right) to basal (bottom left) regions along microtubule filaments in a live late 16-cell stage mouse embryo expressing RNA (magenta) and eGFP-MAP2c (teal). Playback of inset (left side) indicates separate RNA foci with white and orange arrowheads. Time is indicated in m:s:ms.

File Name: Supplementary Movie 4
Description: **Identification of two subpopulations with distinct RNA dynamics in outer blastomere of 16-cell stage mouse embryo.** Tracking of RNA foci in outer blastomere of a 16-cell stage live mouse embryo expressing RNA (magenta) and Membrane GFP (white) displays more dynamic RNA foci in apical regions (white arrowheads) compared to less mobile, clustered RNA foci basally. Time is indicated in m:s:ms.

File Name: Supplementary Movie 5
Description: **Basal movement of RNA and LAMP1-3xeGFP positive vesicular-like structures.** Time-series imaging of a developing 16-cell stage mouse embryo shows RNA (red) and LAMP1- 3xeGFP-positive (green) vesicular like structures (pink arrowheads in playback) move simultaneously toward the basal region. BFP-Utrophin used to show membrane borders (white). Playback shows 3D cropped cell of interest. Time is indicated in h:m:s.

File Name: Supplementary Movie 6
Description: **Lysosomes transport RNA foci.** High temporal resolution time-lapse imaging of RNA foci (magenta; white arrowhead in playback) which undergo directional transport led by the lysosome (LysoSensor Green™; teal; yellow arrowhead in playback) in a live 16-cell stage mouse embryo. Direction of transport (grey arrow in playback). Time is indicated in s.ms.

File Name: Supplementary Movie 7
Description: **Real-time translation kinetics of eIF2b using the SunTag system.** Time-lapse of a live 16-cell stage mouse embryo expressing PCP-2xmCherry (magenta), scFVsfGFP (green)

and SunTag-eIF2b-PP7. Time is indicated in s:ms.

File Name: Supplementary Movie 8
Description: **RNA is evenly inherited by sister cells following symmetric cell divisions from the 16- to 32-cell stage.** Time-lapse of a live mouse embryo expressing RNA (magenta) and Membrane GFP (white) as it divides from the 16- to 32- cell stage demonstrates RNA localised in basal regions is equally inherited during symmetric cell divisions. Playback shows segmented and masked dividing cell which gives rise to two outer sister cells (grey arrowheads). Time is indicated in h:m.

File Name: Supplementary Movie 9
Description: **RNA is unevenly inherited by sister cells following asymmetric cell divisions from the 16- to 32-cell stage.** Time-lapse imaging of a dividing 16- to 32- cell stage mouse embryo expressing RNA (magenta) and Membrane-GFP (white). RNA localised in basal regions is asymmetrically inherited during asymmetric cell divisions (white arrowheads), giving rise to blastomeres in the inside of the embryo (orange arrowheads). Blastomeres inheriting the lesser portion of RNA remain on the outside of the embryo (grey arrowh eads). Time is indicated in h:m.

File Name: Supplementary Movie 10
Description: **Outer sister cells inherit higher ER content following asymmetric cell divisions from the 16- to 32-cell stage.** Time-lapse imaging of a live mouse embryo expressing Emerald-Sec61β (teal) and BFP-Utrophin (white) as it divides from the 16- to 32-cell stage. White arrowheads indicate cells which divide asymmetrically with enriched apical endoplasmic reticulum (ER). Following cell division outer cells (grey arrowheads) inherit the apically localised ER whereas inner cells (orange arrowheads) display a reduced ER fluorescence intensity. The second play back shows segmented and masked 3D cell of interest. Time is indicated in h:m:s.
