# Supplementary material for: Apicobasal RNA asymmetries regulate cell fate in the early mouse embryo
Source: Nat Commun. 2023 May 30;14:2909. doi: 10.1038/s41467-023-38436-2 (PMC10229589; doi:10.1038/s41467-023-38436-2)
Supplement: Supplementary file 14 — Reporting Summary [file 41467_2023_38436_MOESM14_ESM.pdf]

nature portfolio

Corresponding author(s): Jennifer Zenker

Last updated by author(s): Apr 17, 2023

Reporting Summary

Nature Portfolio wishes to improve the reproducibility of the work that we publish. This form provides structure for consistency and transparency in reporting. For further information on Nature Portfolio policies, see our Editorial Policies and the Editorial Policy Checklist.

Statistics

For all statistical analyses, confirm that the following items are present in the figure legend, table legend, main text, or Methods section.

n/a	Confirmed	
☐	☒	The exact sample size (*n*) for each experimental group/condition, given as a discrete number and unit of measurement
☐	☒	A statement on whether measurements were taken from distinct samples or whether the same sample was measured repeatedly
☐	☒	The statistical test(s) used AND whether they are one- or two-sided<br>*Only common tests should be described solely by name; describe more complex techniques in the Methods section.*
☒	☐	A description of all covariates tested
☒	☐	A description of any assumptions or corrections, such as tests of normality and adjustment for multiple comparisons
☐	☒	A full description of the statistical parameters including central tendency (e.g. means) or other basic estimates (e.g. regression coefficient) AND variation (e.g. standard deviation) or associated estimates of uncertainty (e.g. confidence intervals)
☐	☒	For null hypothesis testing, the test statistic (e.g. *F*, *t*, *r*) with confidence intervals, effect sizes, degrees of freedom and *P* value noted<br>*Give P values as exact values whenever suitable.*
☒	☐	For Bayesian analysis, information on the choice of priors and Markov chain Monte Carlo settings
☒	☐	For hierarchical and complex designs, identification of the appropriate level for tests and full reporting of outcomes
☒	☐	Estimates of effect sizes (e.g. Cohen's *d*, Pearson's *r*), indicating how they were calculated

Our web collection on statistics for biologists contains articles on many of the points above.

Software and code

Policy information about availability of computer code

Data collection	ZEN Black (2010), ZEN Blue 3.3, 3.4 and 3.5, LAS X, Bowtie2 v2.3.4.1, sambamba v0.6.7, samtools view v1.12, Picard v2.18.0, GNU parallel v20161222, deepTools bamCoverage v3.4.3
Data analysis	ZEN Blue 3.4 software, Imaris 7.4.2 and 9.5.1 software (Bitplane AG), ImageJ/Fiji v1.52d, GraphPad Prism 9, Excel 2019, Adobe Illustrator 2023 v.27.4, Adobe Photoshop 2021, UCSC genome browser

For manuscripts utilizing custom algorithms or software that are central to the research but not yet described in published literature, software must be made available to editors and reviewers. We strongly encourage code deposition in a community repository (e.g. GitHub). See the Nature Portfolio guidelines for submitting code & software for further information.

Data

Policy information about availability of data

All manuscripts must include a data availability statement. This statement should provide the following information, where applicable:

- Accession codes, unique identifiers, or web links for publicly available datasets
- A description of any restrictions on data availability
- For clinical datasets or third party data, please ensure that the statement adheres to our policy

The authors declare that the data supporting the findings of this study are available within the article and Supplementary Information files. Supplementary movies are linked to this article. Source Data are provided with this paper. All remaining data will be available from the corresponding author upon reasonable request.

Human research participants

Policy information about studies involving human research participants and Sex and Gender in Research.

Reporting on sex and gender	n/a
Population characteristics	n/a
Recruitment	n/a
Ethics oversight	n/a

Note that full information on the approval of the study protocol must also be provided in the manuscript.

Field-specific reporting

Please select the one below that is the best fit for your research. If you are not sure, read the appropriate sections before making your selection.

☒ Life sciences ☐ Behavioural & social sciences ☐ Ecological, evolutionary & environmental sciences

For a reference copy of the document with all sections, see nature.com/documents/nr-reporting-summary-flat.pdf

Life sciences study design

All studies must disclose on these points even when the disclosure is negative.

Sample size	At least 3 embryos per experiment, with at least 2 cells per embryo.<br>This work used preimplantation mouse embryo which are difficult to obtain, numbers to be used are limited and controlled by animal ethics and litter size varies every round. Every experiment were started with at least 20 embryos. However, a number of isolated 1-cell stage embryo may not be fertilised and won't develop, others may lyse after microinjection and others may not express sufficiently the microinjected fluorescently-tagged RNA construct, reducing the number of embryos suitable for an experiment.
Data exclusions	Only data of high quality embryos were used (embryos reaching each cell stage at the expected time point, no signs of blebbing or lysing, expected morphology of cells).
Replication	Data were verified in at least 3 embryos.
Randomization	Embryos were randomly assigned to different experimental groups on the day of microinjection or treatment.
Blinding	Embryos were treated in a blinded fashion.

Reporting for specific materials, systems and methods

We require information from authors about some types of materials, experimental systems and methods used in many studies. Here, indicate whether each material, system or method listed is relevant to your study. If you are not sure if a list item applies to your research, read the appropriate section before selecting a response.

Materials & experimental systems

n/a	Involved in the study	
☐	☒	Antibodies
☒	☐	Eukaryotic cell lines
☒	☐	Palaeontology and archaeology
☐	☒	Animals and other organisms
☒	☐	Clinical data
☒	☐	Dual use research of concern

Methods

n/a	Involved in the study	
☐	☒	ChIP-seq
☒	☐	Flow cytometry
☒	☐	MRI-based neuroimaging

Antibodies

Antibodies used	beta-tubulin at 1:1000 (Abcam, ab21057), ab21057 has been referenced in 31 publications<br>CAMSAP3 at 1:500 (Aviva OAAB13856),<br>CAMSAP3 at 1:500, Home-made by Masatoshi Takeichi, Tanaka et al.,2012

Ninein clone 79-160-7 at 1:500 (MilliPore, MABT29), Kashihara et al., 2018
LAMP1 clone 1D4B at 1:1000 (Abcam, ab25245), ab25245 has been referenced in 132 publications
alpha-tubulin clone DM1A at 1:1000 (Sigma, T6199), T6199 has been referenced in >2000 publications
acetylated alpha-tubulin clone 6-11B-1 at 1:1000 (Sigma, 6793), 6793 has been referenced in >1000 publications
Sec61beta clone D5Q1W at 1:500 (Cell Signalling, 14648), 14684 has been referenced in at least 6 publications
CDX2 at 1:100 (Abcam, ab88129), discontinued, Rispal et aal. 2019
CDX2 clone CDX2-88 at 1:100 (Abcam, ab157524), discontinued, Haller et al., 2019
Oct3/4 clone C-10 at 1:100 (Santa Cruz, sc5279), sc5279 has been referenced in >2000 publications
SOX2 clone Btjce at 1:200 (ThermoFisher, 14-9811-82), this antibody was verified by Knockdown to ensure that the antibody binds to the antigen stated, referenced in 50 publications
Puromycin clone 12D10 at 1:300 (Merck, MABE343)
RPL10A clone ARC2930 at 1:1000 (ABclonal, A20944)

goat anti-rat IgG AF647 (Invitrogen, A21247),
donkey anti-mouse IgG AF488 (Invitrogen, A21202),
donkey anti-rabbit IgG AF586 Invitrogen, A10042),
donkey anti-rabbit IgG AF488 (Invitrogen, A21206),
goat anti-rat IgG AF488 (Invitrogen, A11006),
donkey anti-goat IgG AF647 (Invitrogen, A32849),
all at 1:500

Validation

All antibodies were tested on cell lines were protein was known to be expressed (P19 cells, HEK293 cells), and a control immunostaining without primary antibody was performed on embryos. Additional verification statements are included above.

Animals and other research organisms

Policy information about studies involving animals; ARRIVE guidelines recommended for reporting animal research, and Sex and Gender in Research

Laboratory animals

Animals were housed in pathogen-free animal house conditions at 23 C in a 12 h light/dark cycle at the animal facility (Monash Animal Research Platform). 8- to 9-week old FVB/N wild-type female mice, mated with mated with FVB/N stud males (6- to 20 weeks).

Wild animals

No wild animals were used in this study.

Reporting on sex

Sex was not considered as work was performed with preimplantation mouse embryos, when sex is not yet determined.

Field-collected samples

No field collected samples were used in this study.

Ethics oversight

Experiments were approved by the Monash Animal Ethics Committee (MARP-3) under animal ethics number 19143.

Note that full information on the approval of the study protocol must also be provided in the manuscript.

ChIP-seq

Data deposition

☒ Confirm that both raw and final processed data have been deposited in a public database such as GEO.

☐ Confirm that you have deposited or provided access to graph files (e.g. BED files) for the called peaks.

Data access links
May remain private before publication.

n/a; we used publicly available ChIPSeq data (Adachi et al. 2013; Amin et al., 2016; Matsuda et al., 2017; Boroviak et al., 2018) but did not generate them.

Files in database submission

n/a; we used publiclyavailable ChIPSeq data (Adachi et al. 2013; Amin et al., 2016; Matsuda et al., 2017; Boroviak et al., 2018) but did not generate them.

Genome browser session
(e.g. UCSC)

n/a; we used publicly available ChIPSeq data (Adachi et al. 2013; Amin et al., 2016; Matsuda et al., 2017; Boroviak et al., 2018) but did not generate them.

Methodology

Replicates

n/a; we used publicly available ChIPSeq data (Adachi et al. 2013; Amin et al., 2016; Matsuda et al., 2017; Boroviak et al., 2018) but did not generate them.

Sequencing depth

n/a; we used publicly available ChIPSeq data (Adachi et al. 2013; Amin et al., 2016; Matsuda et al., 2017; Boroviak et al., 2018) but did not generate them.

Antibodies

n/a; we used publicly available ChIPSeq data (Adachi et al. 2013; Amin et al., 2016; Matsuda et al., 2017; Boroviak et al., 2018) but did not generate them.

Peak calling parameters

Single end fastq files were obtained from the publicly available data71-73 and were quality trimmed with trimmomatic vX using

Peak calling parameters	parameter SE. The fastq files were then aligned with Bowtie2 vX using parameters ..xxx. The output sam files were converted into bam files, and were sorted and indexed using sambamba with default parameters. Bam files were then filtered for blacklisted regions using samtools, the duplicates were removed using Picard, and low quality reads were removed using samtools view with parameter -q 10. Peaks were then called for each study and protein using macs2 with default parameters for a mouse genome (replicates were used together if there were any), dataxxx did not have an input file for parameter -c. Nearest genes were annotated with Homer vX. deepTools vX was used to generate genome coverage tracks (bigWig). Datasets used: 0.1242/dev.143479 (GSM1924746 sox2 chip, GSM1924743); 10.1016/j.celrep.2016.11.069 (GSM2253707 and GSM2253708 (rep 1 2 Cdx2 EPISC); 10.1016/j.molcel.2013.09.002 (SRR1068150 sox2 chip, SRR1068152 cdx2 chip, input - SRR1068154).
Data quality	Single end fastq files were obtained from the publicly available data71-73 and were quality trimmed with trimmomatic vX using parameter SE. The fastq files were then aligned with Bowtie2 vX using parameters ..xxx. The output sam files were converted into bam files, and were sorted and indexed using sambamba with default parameters. Bam files were then filtered for blacklisted regions using samtools, the duplicates were removed using Picard, and low quality reads were removed using samtools view with parameter -q 10. Peaks were then called for each study and protein using macs2 with default parameters for a mouse genome (replicates were used together if there were any), dataxxx did not have an input file for parameter -c. Nearest genes were annotated with Homer vX. deepTools vX was used to generate genome coverage tracks (bigWig). Datasets used: 0.1242/dev.143479 (GSM1924746 sox2 chip, GSM1924743); 10.1016/j.celrep.2016.11.069 (GSM2253707 and GSM2253708 (rep 1 2 Cdx2 EPISC); 10.1016/j.molcel.2013.09.002 (SRR1068150 sox2 chip, SRR1068152 cdx2 chip, input - SRR1068154).
Software	Pre-calculated z-scores for expression throughout preimplantation embryo development were utilised from Boroviak et al., scRNA-seq data74. R base functions, custom script, and ggplot2 were used for data exploration and visualisation of the ChIP-seq targets, UpSetR vX was used to plot the overlaps. UCSC genome browser was used for visualiation of the bigWig tracks.
